# Influence of distance, area, and cultural context in active commuting: Continental and insular children

**DOI:** 10.1371/journal.pone.0213159

**Published:** 2019-03-05

**Authors:** Fernando Rodríguez-Rodríguez, Oscar Pakomio Jara, Norman Macmillan Kuthe, Manuel Herrador-Colmenero, Robinson Ramírez-Vélez, Palma Chillón

**Affiliations:** 1 Grupo IRyS, School of Physical Education, Pontificia Universidad Católica de Valparaíso, Valparaíso, Chile; 2 Grupo de Investigación PROFITH "PROmoting FITness and Health THrough physical activity". Departamento de Educación Física y Deportiva, Facultad de Ciencias del Deporte, Universidad de Granada, Granada, Spain; 3 La Inmaculada Teacher Training Centre, University of Granada, Granada, Spain; 4 Department of Health Sciences, Public University of Navarra, Navarrabiomed, Pamplona, Navarra, Spain; Tongji University, CHINA

## Abstract

Commuting by walking or cycling is a way to increase physical activity levels. The objective of this article was to determine the modes of commuting to school and the distance and time of the way to school among children from Easter Island and from the mainland (Valparaíso), in Chile. A total of 666 children and adolescents aged 10 to 18 years old (208 from Easter Island and 458 from Valparaíso) participated and completed a valid questionnaire including data about age, gender, usual commuting mode to and from school, distance, and travel time. There are important differences in the mode of commuting between students of Valparaíso and Easter Island. Private transport is more commonly used in Valparaíso than in Easter Island (p<0.001). Furthermore, it was observed that cycling and public transportation are not used as mode of commuting in Valparaíso and Easter Island respectively. Students from Easter Island, who travel more distance and during more time, are more active than students from Valparaíso (going 24.8% and 17.6%; from: 61% and 28.8% respectively). This situation is influenced by the geographic context of the island, the distances from home to school, and the type of commuting, which fosters the level of active commuting. On the other hand, the passive modes of commuting to school are higher in the mainland urban setting of Valparaíso. It is necessary to study the diverse contexts of the Easter Island population, but, for now, the rural setting of Easter Island seems to be associated with a greater level of active commuting to school.

## Introduction

Active school transport can be defined as the type of commuting by which children or adolescents cover the distance between home and school using modes that do not involve motorized vehicles, such as walking or cycling [1, 2]. On the other hand, passive commuting refers to the use of motorized vehicles as a mode of transport, such as by car, bus, metro, train, motorcycle, or others [3]. Where passive commuting is concerned, commuting by public transport (i.e. bus, metro or train) is more active than by private transport (i.e. car or motorcycle), because to reach the bus, metro or train stops, it commonly requires walking. Public transport can thus be considered a mixed mode of transport [4].

Active commuting to school provides an opportunity to increase levels of physical activity, and to improve the physical fitness and the health status of these students [5]. Children and adolescents that commute actively are able to increase the time dedicated to physical activity between 5–37 minutes/day [6]. More specifically, regarding it is benefits, Andersen [7] observed that children aged 9.7 ± 0.5 years old that commuted actively presented lower levels of body fat and a lower likelihood of acquiring cardiac diseases. Recently, Ramírez-Vélez [8], showed that cycling to school regularly may be associated with better physical fitness and a lower incidence of metabolic syndrome than passive transport, especially in girls. Likewise, active commuting has been associated with a better cognitive performance in adolescents aged 13–18.5 years old [9] and a reduction in stress in students aged 10–14 years old during school hours [10].

In spite of these benefits, the frequency of active commuting to school has drastically decreased in the last thirty years in countries like the United States [11], United Kingdom [12], Canada [13], Australia [14], New Zealand [15], and Spain [16]. Previous data from South America point out that Mexican teenagers (10–14 years old) walked to school at a rate of 68%, and a 2% cycled to school [17], while Colombian children (10.5 ± 0.6 years old) and Brazilian children (10.5 ± 0.5 years old) showed active commuting rates (walking or by bike) of 71.5% and 40% respectively [18]. A lower rate of active commuting was found in Ecuador, where a 14.2% of children and a 20% of adolescents were active [19]. In Chile, among the rural adolescents (12–13 years old), around a 22.9% and a 28.5% of them commuted actively to and from school respectively [20], while only an 11.0% of children (10.6 years old) and a 24.8% of adolescents (13.9 years old) from urban areas walked to school [21]. The behavior of active commuting is very contextual-specific because of the diversity of cultural and geographic factors.

Previous studies have proven that the main barrier for commuting actively to school is the distance [22, 23]. In Europe, it has been observed that Belgian children aged 11–12 years old commute 1.5 km walking, while teenagers aged 17–18 years old commute walking up to 2 km [24, 25]. Commuting thresholds have also been established in British students. At the age of 10, the threshold distance to walk to school is 1.4 km, increasing up to 1.6 km in children aged 11 years and 3 km in adolescents aged 14 years [26]. In Spain, the threshold distance to walk to school was 0.88km for elementary school children (7–12 years old) and 1.35 km for high school adolescents (13–18 years old) [27]. It is an overall issue that the farther children live from school, the less likely it is for them to commute actively. However, the walkable distance from home to school seems to be another contextual-specific issue, regarding the urban planning and the cultural perception of a walkable distance. For example, the proportion of active commuting to school within a distance of 3 km is 15% in the United States [28], whereas in Finland it is 75% for the same distance [29].

Likewise, the ethnic origin of the participants may present differences in the modes of commuting, as well as in the level of physical activity of students [30]. Chile possesses ethnic and cultural differences. For instance, Easter Island (Rapa Nui) has particular characteristics because it is far away from the mainland (3700 km), the natural environment based on rural areas, and its cultural origins from Polynesia (Maori origin), with different customs and a folklore based on dances and ancestral competitions. Traditionally, both physical activity and healthy living have been naturally promoted [31]. Research in physical activity on the island is scarce, however, scientific evidence has indicated a low incidence of obesity (24%) and more children with normal weight (56%) in relation to the current national average of childhood obesity and normal weight in mainland (25.3% and 41.8% respectively). Additionally, they practice more hours of physical education per week than their counterparts in the mainland, which is common among the Rapa Nui population [32]. These results suggest that currently there may be differences between children and adolescents on Easter Island and on the mainland regarding healthy lifestyles, such as the mode of commuting to school.

Therefore, the objective of this study was to determine and compare the modes of commuting to and from school among children from Easter Island (island territory) and Valparaíso (mainland territory) in Chile.

## Materials and methods

### Participants and design

A total of 666 Chilean students (children aged 9–11 years old and adolescents aged 12–18 years old) who agreed to participate in this study. From the total, 208 came from 2 different schools in the province of Easter Island (122 children and 86 adolescents, of a total universe of 667 schoolchildren), and 458 came from 3 different schools in Valparaíso (176 children and 282 adolescents, of a total universe of 1,500 schoolchildren). This was a cross-sectional study with a non-probability sample of volunteer students.

### Instruments and procedure

The questionnaire used was the Chilean version of the previous valid Spanish questionnaire designed for measuring the modes and frequency of commuting to and from school [33] (http://profith.ugr.es/paco). Two questions were asked about the usual mode of commuting to and from school. Each question provided the following answers: walk, cycle, car, motorcycle, school bus, public bus, metro/train, or other (in this case, the mode was required). In addition, the modes were classified to build two variables: Active (walk, cycle) vs Passive (others mode, no walk or cycle); and Active (walk, cycle) vs Private transport (car, motorcycle, school bus) vs Public transport (public bus, metro/train).

Moreover, questions were asked regarding age, gender, distance, and time from home to school. Distances were measured in kilometers, and they were divided into 5 categories (0–0.5 km, 0.5–1 km, 1–2 km, 2–3 km, 3–5 km, and >5 km). Time was categorized into 0–15 minutes, 16–30 minutes, 31–60 minutes, and >60 minutes. A test-retest reliability analysis of questions about the mode of commuting to school, distance, and time in the Chilean version was done with Kappa statistic values (2 questionnaires were completed separated by 7 days). The mode of commuting to and from school shows a high reliability (Kappa > 0.85), whereas commuting distance shows a moderate reliability (Kappa > 0.69).

The participants had between 15 and 50 minutes to complete this self-reported questionnaire in classroom. They were accompanied and helped by the researchers and the Physical Education teacher. The questionnaires were completed by students in grades 5–8 in Elementary and Middle School (children aged 9–11 years old) and 9–11 in High School (adolescents aged 12–18 years old). The information collected from children and adolescents was provided voluntarily and with written consent and signed by the parents, who were informed about the types of the questions, as well as the objectives and the confidentiality of the results. Therefore, all participants gave informed consent for participating in the study, following the rules and approved by Ethics Committee of the Pontificia Universidad Católica de Valparaíso (code: CCF02052017) and following ethical standards that were in accordance with the Declaration of Helsinki 2004.

### Statistical analysis

A descriptive statistical analysis was performed by obtaining means and standard deviations for continuous variables and percentages for categorical variables, according to sex and age group. Chi-square analyses were performed to determine the differences between the students of Valparaiso and Easter Island for the two mode of commuting variables created: Passive vs Active, and Passive vs Public transportation vs Private transportation.

To perform the statistical analyses, we used the software IBM SPSS Statistics version 21, establishing a level of trust of 95% and a statistical significance of p<0.05.

## Results

Table 1 shows the descriptive data of the participants regarding the place of origin in Chile (i.e., Easter Island vs. Valparaiso). Concerning the distance from home to school, 70% of the students from Easter Island and 52% from Valparaíso commuted a distance between 0 to 3 km. In relation to the time spent commuting from home to school, 70% of the students from Easter Island and 39% from Valparaíso took less than 15 minutes to commute from home to school.

**Table 1:** Descriptive data of the participants.

	Overall n (%) (n = 666)	Valparaíso n (%) (n = 458)	Easter Island n (%) (n = 208)
**Sociodemographic factors, % (n)**			
*Gender*			
Male	347 (52.1)	231 (50.4)	116 (55.8)
Female	319 (47.9)	227 (49.6)	92 (44.2)
*Age groups*			
Children (9–11 y)	298 (44.7)	176 (38.4)	122 (58.4)
Adolescents (12–18 y)	369 (55.3)	282 (61.6)	87 (41.6)
**Distance to school groups, % (n)**			
<0.5 km	88 (13.9)	63 (14.9)	25 (12.0)
0.5–1 km	94 (14.9)	60 (14.2)	34 (16.3)
1–2 km	101 (16.0)	58 (13.7)	43 (20.6)
2–3 km	81 (12.8)	37 (8.7)	44 (21.1)
3–5 km	79 (12.5)	49 (11.6)	30 (14.4)
>5 km	189 (29.9)	156 (36.9)	33 (15.8)
**Commuting Time, % (n)**			
<15 min	326 (48.6)	180 (39.3)	146 (69.9)
15–30 min	210 (31.3)	161 (35.2)	49 (23.4)
30–60 min	74 (11.0)	64 (14.0)	10 (4.8)
>60 min	21 (3.1)	17 (3.7)	4 (1.9)

Table 2 specifies the mode of commuting of students from Easter Island and Valparaíso. On the way to school, commuting by car is prevalent in both territories, but it is significantly higher on Easter Island (p = 0.001). In Easter Island, public transportation such as public bus, school bus, metro/train is non-existent (0%). However, commuting by bicycle is ten times higher in Easter Island than in Valparaíso (10% and 0% respectively), since cycling for commuting is null in the mainland. From school to home, walking values of Easter Island double those of Valparaíso (51.2% and 24.8%).

**Table 2:** Commute mode to and from school in Valparaíso and Easter Island.

	*Going to School*					*From school*				
*Commuting*	Valparaíso		Easter Island		*p value*	Valparaíso		Easter Island		*p value*
*Mode*	n	(%)	n	(%)		n	(%)	n	(%)	
*Walking*	74	(17.6)	35	(16.8)	0.455	104	(24.8)	106	(51.2)	<0.001
*Cycling*	0	(0.0)	25	(12.0)	^a^	0	(0.0)	22	(10.6)	^a^
*Car*	236	(56.1)	143	(68.8)	0.001	148	(35.3)	79	(38.2)	0.271
*Motorcycle*	5	(1.2)	5	(2.4)	0.206	27	(6.4)	0	(0.0)	^a^
*School Bus*	27	(6.4)	0	(0.0)	^a^	33	(7.9)	0	(0.0)	^a^
*Public Bus*	18	(4.3)	0	(0.0)	^b^	100	(23.9)	0	(0.0)	^b^
*Metro/train*	61	(14.5)	0	(0.0)	^b^	6	(1.4)	0	(0.0)	^b^

Statistical significance in a Chi-Square test with a value of p<0.05.

^a^ p-value was not calculated, because there was no prevalence.

^b^ p-value was not calculated, because there no exist commuting mode in Easter Island

**Fig 1:**
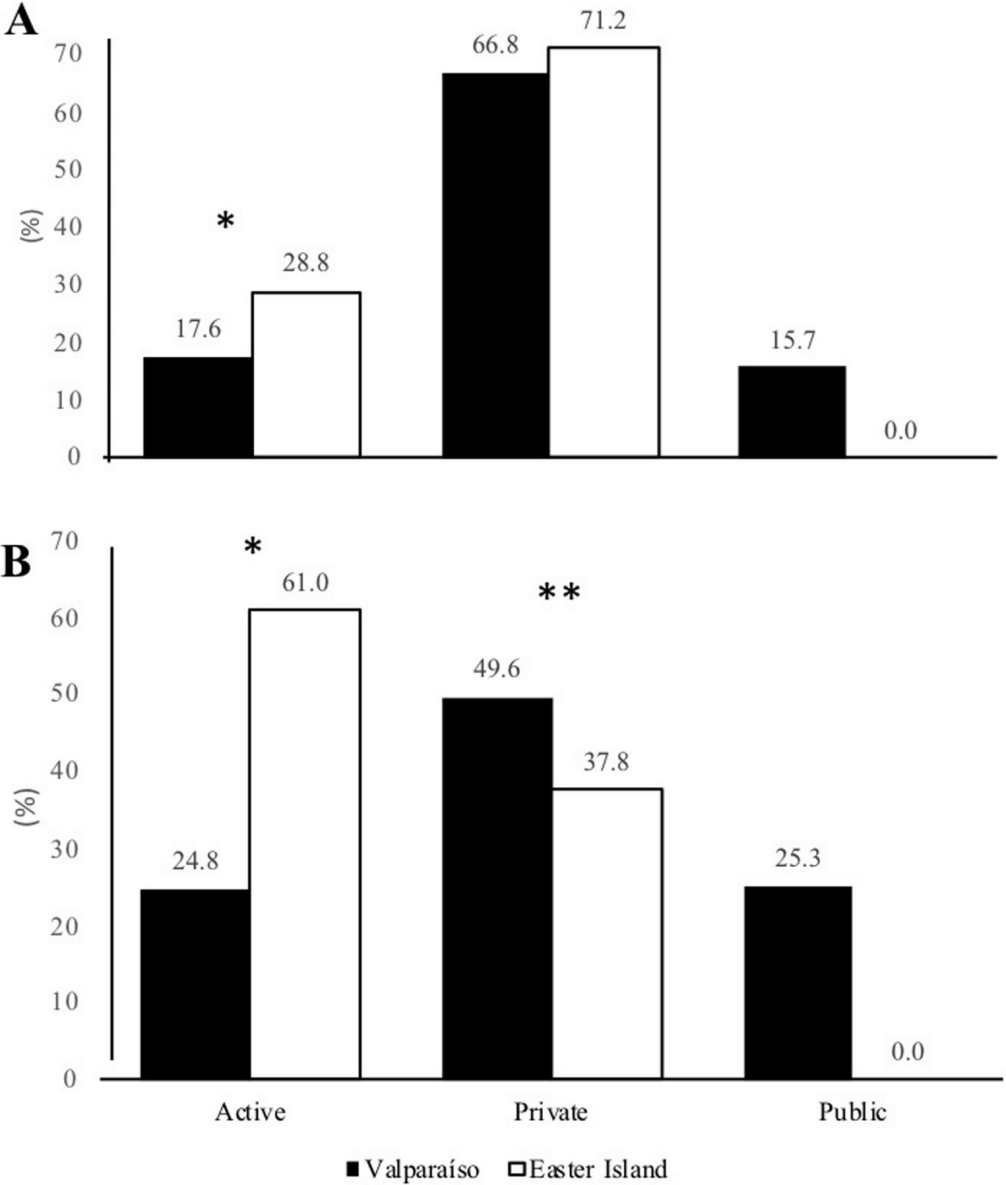
Comparative modes of commuting, where: Active is walking and cycling. Private is car, motorcycle and school bus, Public is public bus and metro/train. (A) Going to school; (B): From school. * Indicates statistical significance at the p<0.001 level. ** Indicates statistical significance at the p<0.01 level. In Public, p-value was not calculated, because there was no prevalence.

Fig 1 shows the modes of commuting regarding the categories of Active, Private, and Public transport. There is a higher prevalence of active commuting to and from school in students from Easter Island compared to students from Valparaíso (p<0.001). In relation to private commuting to and from school, the values are higher in students from Valparaíso compared to students from Easter Island (p<0.001). There was no use of public transport in Easter Island.

**Fig 2:**
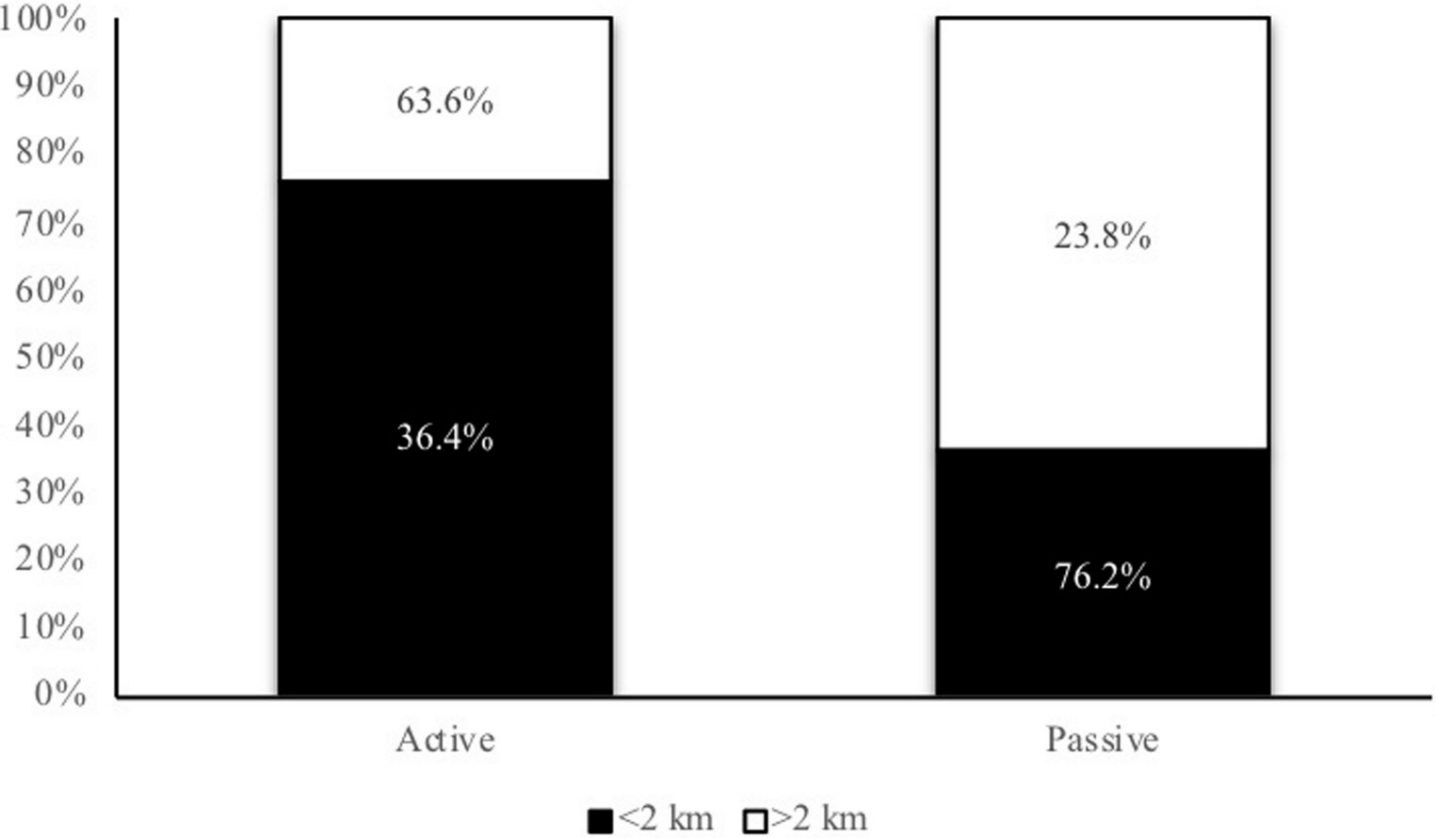
Comparative modes of commuting, where: Active is walking and cycling. Passive is car, motorcycle, school bus, public bus, and metro/train. Statistical significance at the p<0.01 level, between Passive and Active commuting adjusted to distance.

Comparing active children and adolescents in relation to the distance traveled (Fig 2), data show that when the distance commuted is <2 km there is a high percentage of who commute actively (76.1%). In contrast, the distance range of >2 km presents a greater value of children who commute passively (63.6%).

## Discussion

In the current study, we observed that children and adolescents from Easter Island walked and cycled more, being more active than their counterparts of Valparaiso. In Easter Island, the students cover minor distance and spend less time for commuting than those of Valparaiso. However, cars are the main mode of commuting in both groups.

### Prevalence of active commuting to school in rural and urban areas

A variety of studies exist that have focused on analyzing the levels of active commuting to school among children and adolescents on a worldwide level [17–29]. These studies show a greater level of active commuting in adolescents than in children, being higher on the way to home from school, which agree with the results obtained in the present study. Despite the fact that these similarities were observed among the students of Valparaíso and Easter Island, there are marked differences in the commuting modes, which make the students of Easter Island more active. In this respect, the population projections made by the National Institute of Statistics of Chile (INE) point out that the population in Valparaíso reached 295,927 residents in 2017 (density: 94.1 inhabitants/km^2^), who 0.3% are in rural areas and 99.7% in urban areas (16,396 km^2^ of surface), whereas Easter Island only reached 7,750 (density: 47.3 inhabitants/km^2^) [34], who 15.7% belong to rural areas and 84,7% to urban areas (163.3 km^2^ of surface). This fact allows an urban and rural context to be distinguished. A North American study suggests that children from rural areas are more likely to be active commuters than urban children [35]. On the other hand, when comparing children from urban and rural areas from Brazil, data shows that children from urban areas present higher levels of active commuting to school than children from rural areas [36].

Additionally, children who live in rural areas may have more opportunities for active play or active commuting and more limited access to technologies such as the Internet [36, 37], which is the case of Easter Island, where there is no cable television and only three national channels are broadcast. Likewise, students from urban areas have started to increase their sedentary behavior, which can be associated with the increase in time spent in front of screens [38]. This behavior can be defined as the total amount of time spent watching television, using computers or playing video games, a phenomenon that is very common among young people [39]. These behaviors, especially the time spent watching television, has also been associated with a higher risk in children of being overweight or obese [40, 41, 42].

An important result is that no participant used the bike to get to school in Valparaíso. In addition, one must consider the geographical context of the region of Valparaíso, that has important inconveniences such as long distances from home to school and a geography with differences between the hills and the coast. Moreover, the scarce availability of bicycle paths (only 2 km), which disfavours this mode of transport [21]. For other hand, the urban area of Easter Island that is flat and less cars circulate, which can be associated with the highest use of the bicycle (around 10%).

**Fig 3:**
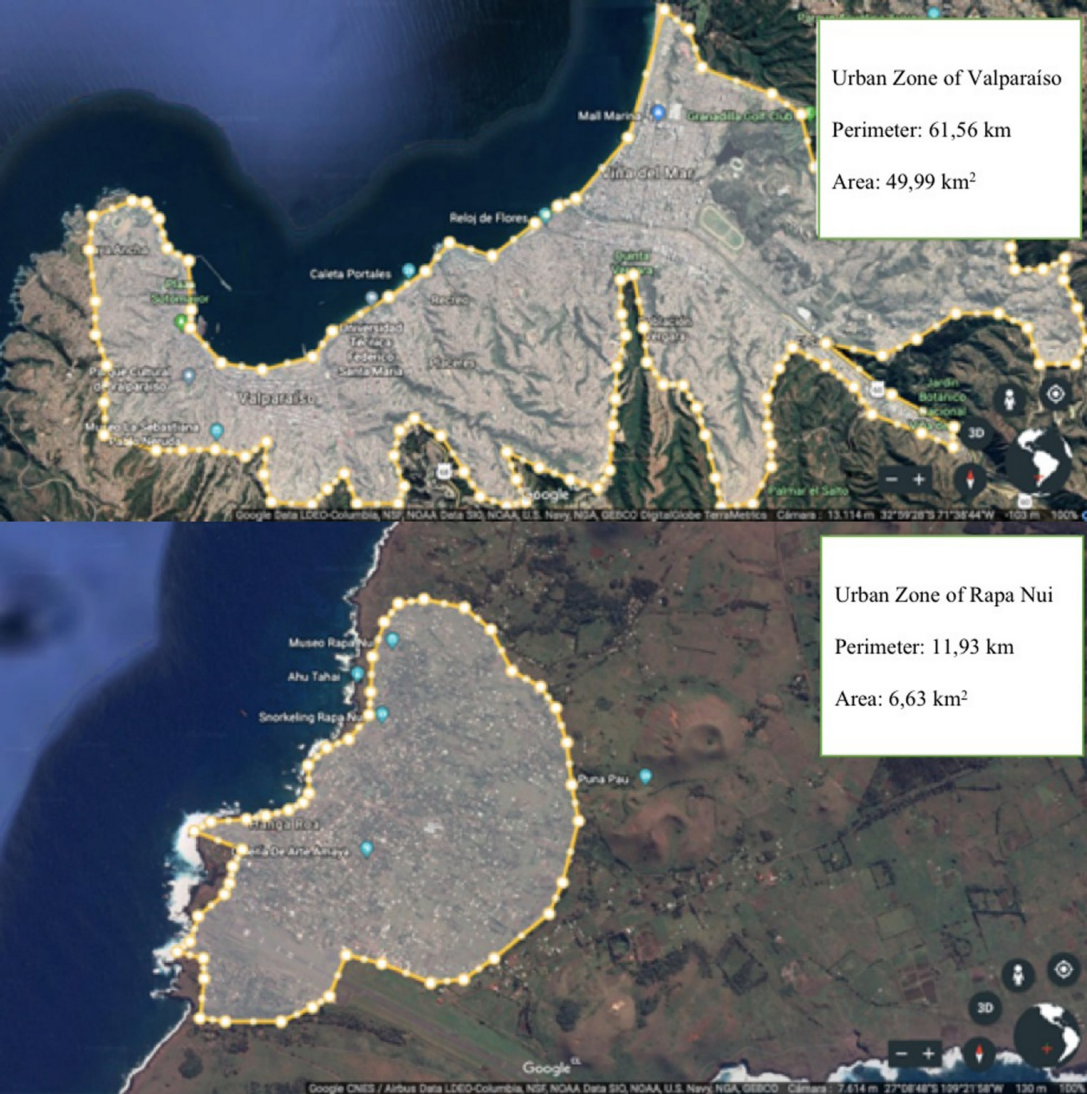
Urban areas of both cities (Rapa Nui island and Valparaiso in mainland) belonging to the same region.

A study performed in California suggests that the independence that students gain as they grow up in rural areas, combined with the increase in the use of public transport to get to school provides, in turn, an opportunity to increase the level of physical activity of this population [43]. In urban students from Valparaíso, the use of public transportation when returning from school increased significantly (p<0.05). Nevertheless, the fact that this type of commuting mode almost does not exist in Easter Island (only taxi) due to the small area of the island and the concentration of people in the urban area that does not exceed 7 km^2^. This could influence the significant increase in active commuting (i.e. walking or cycling) in both going to school and going back from school, especially if the commuting distances are smaller, unlike Valparaiso with almost 50 km^2^ of urban area, which strongly impacts the distances that must be traveled (Fig 3), decreasing the options of active commuting.

### Distance of active commuting to school

The distance covered for commuting to school in Easter Island is lower (barely 15% of students cover more than 5 km) than in Valparaíso (33.3% of students commute more than 5 km). The vast distances between the home of each student and their school in the Chilean urban setting are the main limitation in promoting more active commuting to school behaviours.

In urban areas in India, 90% of students between the ages of 11 and 14 years old live less than 5 km from their school, and 36% live less than 1 km. Greater distances to school were strongly associated with the use of passive modes of transport. Children that lived close to their school were much more likely to walk (56%) or go by bicycle (6%) [44]. An Australian study affirms that the greater the distance between the home and school was, the lower the percentage of students that commuted actively every day was [45]. Furthermore, students that lived less than 0.75 km from school were more active than those who lived farther than 0.75 km. Another Spanish study in a rural setting defined a distance of 0.8 km as the walking cut-off point for children [46]. Furthermore, other researchers have established differences between the cut-off distance for children and adolescents, from 0.88 km to 1.35 km respectively [47]. All these results have indicated that long distances from home to school could be a major limitation in promoting the daily active commuting of the student population to and from school, which can also be observed in our results. Long distances to school imply a low likelihood of adopting active transport practices [43], and distances of approximately 2 km are associated with the best physical activity related to active commuting [47].

Given that the schools are far away from residential areas, the adoption of policies to reduce distances and promote active commuting would reduce the gap between European and Chilean students. Smaller distances to school are associated with the enrollment of students in the closest school. This makes it necessary for policies to incentivize enrollment in the nearest school, which could increase the level of active transport and contribute to tackling the impacts that come from physical inactivity among young people.

### Cultural context

The part of the population on Easter Island of Maori origin is one of the few cultures that keeps its original culture intact and maintains the same customs and lifestyle. Other Maori communities, such as the one found in New Zealand has become urbanized enough to the point that almost no difference can be found between their active commuting behavior and that of the European New Zealand population. The significance is barely p = 0.12 with an Odds Ratio of hardly 1.55 between both ethnicities [48], including 2.3% less time in moderate-to-vigorous physical activity than the European New Zealand population [49]. In addition to this, a greater proportion of Maori lives in disadvantaged geographic areas, with lower family incomes and with a formal education that is inferior to that of European countries. This dynamic, in turn, decreases their level of physical activity and increases levels of childhood obesity [50]. This evidence contrasts with the commuting mode on Easter Island, and that is not related to the level of income, because the level of poverty on the island reaches 8%, while in Valparaíso is 15.4% [51]. Another study determined the differences in median income, being $NZ14,800 for Maori compared with $NZ19,800 for European New Zealanders. Despite disparities in income level, the relatively high physical activity level (69% perform >150 min/week), is likely to be related to other social factors [52], that have been specific to culture (horticultural, hunters and fished for food).

Children with parents who perceive their neighbourhood as more connected, and are located closer to school, engaged in higher levels of independent mobility in NZ European, Maori and Samoan population, unlike other contexts, where “traffic danger” was the most common reason for concern [53]. The cohesion of the inhabitants of Easter Island, allows for a better sense of security, due to the proximity of schools, the small urban area and low number of cars. These elements are key, allowing Easter Island to be considered as an example for other communities by the urban organization and adequate environment that favors the active commuting to school.

### Limitations and strengths

This study has limitations such as the low number of total participants, the low heterogeneity of Chilean cities included in the sample, and the distance data that was self-reported. In addition, the statistical analysis performed do not allow to include variables as confounders; for future researching, more variables identifying the two samples should be included to perform more complex statistical regression models [54]. The main strength of the current study is the description of the modes of commuting in Chilean children and adolescents in one of the most important cities, especially on Easter Island. These findings contribute to the particular knowledge of this region of South America and Maori culture. In addition, the reliability of the questionnaire used in the study is also a strength, since it reduces the error in the answers of the students.

## Conclusions

There are important differences in the mode of commuting between students from the urban areas of Valparaíso compared with students from Easter Island, who present higher values of active modes of commuting (i.e. walking and cycling). This situation is influenced by the cultural context of the island, the distances from home to school, and the type of commuting, which fosters the level of active commuting. On the other hand, the passive modes of commuting to school are higher in the mainland urban setting of Valparaíso. Consequently, the rural setting of Easter Island seems to promote a greater level of active commuting to school.

## Supporting information

S1 Data Data support in RAPA NUI. (SAV)

## References

[1] Chillón P, Evenson KR, Vaughn A, Ward DS. A systematic review of interventions for promoting active transportation to school. Int. J. Behav. Nutr. Phys. Act. 2011; 8,10 10.1186/1479-5868-8-10 21320322 PMC3050785

[2] Larouche R. Assessing the health-related outcomes and correlates of active transportation in children and youth. Appl. Physiol. Nutr. Metab. 2014; 39(3): 403.

[3] Villa-González E, Rodríguez-López C, Barranco-Ruiz Y, Cabezas-Arévalo L, Chillón P. Evaluación de la concordancia de dos métodos para determinar la distancia del desplazamiento activo al colegio en escolares. Nutr Hosp. 2016; 33(3): 713–718. 10.20960/nh.283 27513510

[4] Fraser S and Lock K. Cycling for transport and public health: a systematic review of the effect of the environment on cycling. Eur. J. Public Health. 2011; 21(6): 738–743. 10.1093/eurpub/ckq145 20929903

[5] Lubans DR, Boreham CA, Kelly P. The relationship between active travel to school and health-related fitness in children and adolescents: a systematic review. Int. J. Behav. Nutr. Phys. Act. 2011; 8(1): 5. 21269514 10.1186/1479-5868-8-5 PMC3039551

[6] Faulkner GEJ, Buliung RN, Flora PK, Fusco C. Active school transport, physical activity levels and bodyweight of children and youth: A systematic review. Prev. Med. 2009; 48(1): 3–8. 10.1016/j.ypmed.2008.10.017 19014963

[7] Andersen LB, Wedderkopp N, Kristensen P, Moller NC, Froberg K, Cooper AR. Cycling to school and cardiovascular risk factors: a longitudinal study. J Phys Act Health. 2011; 8(8): 1025–1033. 22039135 10.1123/jpah.8.8.1025

[8] Ramírez-Vélez R, García-Hermoso A, Agostinis-Sobrinho C. Cycling to School and Body Composition, Physical Fitness, and Metabolic Syndrome in Children and Adolescents. Journal of Pediatrics. 2017; 188: 57–63. 10.1016/j.jpeds.2017.05.065 28651798

[9] Martínez-Gómez D, Ruiz JR, Gómez-Martínez S, Chillón P, Rey-López P, Díaz L et al Active commuting to school and cognitive performance in adolescents: the AVENA study. JAMA Pedriatr. 2011; 165(4): 300–305. 10.1001/archpediatrics.2010.244 21135316

[10] Lambiase MJ, Barry HM, Roemmich JN. Effect of a simulated active commute to school on cardiovascular stress reactivity. Med. Sci. Sports Exerc. 2010; 42(8): 1609 10.1249/MSS.0b013e3181d0c77b 20139790 PMC2907457

[11] MacDonald NC, Steiner RL, Lee C, Rhoulc T, Zhu X, Yang Y. Impact of the safe routes to school program on walking and bicycling. JAPA. 2014; 80(2): 153–167.

[12] Department for Transport. Transport Statistics Bulletin. National Travel Survey: 2008, London. Available at: http://webarchive.nationalarchives.gov.uk/20100513221215/http://www.dft.gov.uk/adobepdf/162469/221412/221531/223955/32274311/NTS2008.pdf (Accessed 1 November, 2017).

[13] Buliung RN, Mitra R, Faulkner G. Active school transportation in the Greater Toronto Area, Canada: an exploration of trends in space and time (1986–2006). Prev Med. 2009; 48(6): 507–512. 10.1016/j.ypmed.2009.03.001 19272403

[14] Van der Ploeg HP, Merom D, Grace Corpuz G. Trends in Australian children traveling to school 1971–2003: burning petrol or carbohydrates? Prev Med. 2008; 46(1): 60–62. 10.1016/j.ypmed.2007.06.002 17628653

[15] Ministry of Transport. 2008 Household Travel Survey: Comparing Travel Modes. Ministry of Transport, Wellington Available at: http://www.transport.govt.nz/assets/Import/Documents/Public20Transport-y57.pdf (Accessed 1 November, 2017).

[16] Chillón P, Martínez-Gómez D, Ortega FB, Pérez-López IJ, Díaz LE, Veiga OL et al Six-year trend in active commuting to school in Spanish adolescents. Int. J. Behav. Med. 2013; 20(4): 529–537. 10.1007/s12529-012-9267-9 23055026

[17] De Sá TH, Rezende LFMD, Borges MC, Nakamura PM, Anapolsky S, Parra D, et al Prevalence of active transportation among adults in Latin America and the Caribbean: a systematic review of population-based studies. Rev Panam Salud Publica. 2017;41:e35. 10.26633/RPSP.2017.35 PMC6614750 31363356

[18] Sarmiento OL, Lemoine P, Gonzalez SA, Royles KD, Denstel R, Larouche V. et al Relationships between active school transport and adiposity indicators in school-age children from low-, middle-and high-income countries. Int J Obes Suppl. 2015; 5: 107–114. 10.1038/ijosup.2015.27 PMC4850628 27152178

[19] Barranco-Ruiz Y, Guevara-Paz A, Ramírez-Vélez R, Chillón P, Villa-González E. Mode of commuting to school and its association with physical activity and sedentary habits in young Ecuadorian students. Int. J. Environ. Res. Public Health. 2018; 15(12), 2704. 10.3390/ijerph15122704 PMC6313456 30513629

[20] García-Hermoso A, Saavedra JM, Olloquequi J, Ramírez-Vélez R. Associations between the duration of active commuting to school and academic achievement in rural Chilean adolescents. Environ. Health Prev. Med. 2017; 22(1), 31 10.1186/s12199-017-0628-5 29165126 PMC5664915

[21] Rodríguez-Rodríguez F, Cristi-Montero C, Célis-Morales C, Escobar-Gómez D, Chillón P. Impact of Distance on Mode of Active Commuting in Chilean Children and Adolescents. Int. J. Environ. Res. Public Health. 2017; 14(11):1334. 10.3390/ijerph14111334 PMC5707973 29099044

[22] Panter JR, Jones AP, van Sluijs EM. Environmental determinants of active travel in youth: are view and framework for future research. Int. J. Behav. Nutr. Phys. Act. 2008; 5(1): 34. 18573196 10.1186/1479-5868-5-34 PMC2483993

[23] Pont K, Ziviani J, Wadley D. Environmental correlates of children's active transportation: a systematic literature review. Health and Place. 2009; 15(3): 827–840. 10.1016/j.healthplace.2009.02.002 19285904

[24] D’Haese S, DeMeester F, De Bourdeaudhuij I, Deforche D, Cardon G. Criterion distances and environmental correlates of active commuting to school in children. Int. J. Behav. Nutr. Phys. Act. 2011; 8(1): 88. 21831276 10.1186/1479-5868-8-88 PMC3168397

[25] Van Dyck D, De Bourdeaudhuij I, Cardon G. Criterion distances and correlates of active transportation to school in Belgian older adolescents. J. Behav. Nutr. Phys. Act. 2010; 7(1): 87. 10.1186/1479-5868-7-87 PMC3004815 21143868

[26] Chillón P, Panter J, Corder K, Jones A, Van Luijs E. A longitudinal study of the distance that young people walk to school. Health & Place. 2015; 31: 133–137. 25528343 10.1016/j.healthplace.2014.10.013 PMC4315806

[27] Rodríguez-López C, Salas-Fariña ZM, Borges-Cosic M. Valencia J, Herrador-Colmenero M, Medina-Casaubón J, et al Distance from home to school: a main correlate on the mode of commuting to school. Revista Andaluza de Medicina del Deporte. 2015; 8(1): 45–46.

[28] Dentro KN, Beals K, Crouter SE, Eisenmann JC, Mckenzie TL, Pate RR. Results from the United States’ 2014 report card on physical activity for children and youth. J Phys Act Health. 2014; 11(Suppl 1): 105–112. 10.1123/jpah.2014-0184 25426905

[29] Tremblay MS, Gray CE, Akinroye K, Harrington D, Katzmarzyk P, Lambert E, et al Physical activity of children: a global matrix of grades comparing 15 countries. J Phys Act Health. 2014; 11(s1): 113–125. 10.1123/jpah.2014-0177 25426906

[30] Owen CG, Nightingale CM, Rudnicka AR. Travel to school and physical activity levels in 9–10 year-old UK children of different ethnic origin; child heart and health study in England (CHASE). PloS One. 2012; 7(2): e30932 10.1371/journal.pone.0030932 22319596 PMC3272007

[31] Englert S. La tierra de Hotu Matu’a: historia y etnología de la Isla de Pascua. Editorial Universitaria, Santiago 2004.

[32] MacMillan N, Rodríguez F, Páez J. Nutritional status, feeding behavior and physical activity of first grade school children from Chilean Easter Island in the last decade. Rev Chil Nutr. 2016; 43(4): 375–380.

[33] Chillón P, Herrador-Colmenero M, Migueles JH, Cabanas-Sánchez V, Fernández-Santos JR, Veiga OL, et al Convervent validation of a questionnaire to assess the mode and frequency of commuting to and from school. Scand. J. Public Health. 2017; 45(6): 612–620. 10.1177/1403494817718905 30747037

[34] INE. Actualización 2002–2012 y Proyección de Población 2012–2020. 2017. Available at: http://www.inevalparaiso.cl/contenido.aspx?id_contenido=13 (Accessed 1 November, 2017).

[35] Lu W, McKyer ELJ, Lee C, Ory MG, Goodson P, Wang S. Children’s active commuting to school: an interplay of self-efficacy, social economic disadvantage, and environmental characteristics. J. Behav. Nutr. Phys. Act. 2015; 12(1): 29. 10.1186/s12966-015-0190-8 PMC4352543 25889664

[36] Neto FA, Eto FN, Pereira TSS, Carletti L, Molina M. Active and sedentary behaviours in children aged 7 to 10 years old: the urban and rural contexts, Brazil. BMC Public Health. 2014; 14(1): 1174. 25404524 10.1186/1471-2458-14-1174 PMC4251846

[37] Hume C, Salmon J, Veitch J, O'Connell E, Crawford D, Ball K. Socio-demographic characteristics of children experiencing socioeconomic disadvantage who meet physical activity and screen-time recommendations: the READI study. Prev Med. 2012; 54(1): 61–64. 10.1016/j.ypmed.2011.10.019 22079447

[38] Peiró-Velert C, Valencia-Peris A, González LM, García-Massó X, Serra-Añó P, Devís-Devís J. Screen media usage, sleep time and academic performance in adolescents: clustering a self-organizing maps analysis. PLoS One. 2014; 9(6): e99478 10.1371/journal.pone.0099478 24941009 PMC4062405

[39] Guthold R, Cowan M, Autenrieth CS, Kann L, Riley LM. Physical activity and sedentary behavior among schoolchildren: a 34-country comparison. Journal of Pediatrics. 2010; 157(1): 43–49. 10.1016/j.jpeds.2010.01.019 20304415

[40] Laurson KR, Eisenmann JC, Welk GJ, Wickel EE, Gentile DA, Walsh DA. Combined influence of physical activity and screen time on recommendations on childhood overweight. Journal of Pediatrics. 2008; 153(2): 209–214. 10.1016/j.jpeds.2008.02.042 18534231

[41] Morales-Ruán M del C, Hernández-Prado B, Gómez-Acosta LM, Shamah-Levy T, Cuevas-Nasu L. Obesity, overweight, screen time and physical activity in Mexican adolescents. Salud Publica Mex. 2009;51 (Suppl 4): 613–620. 10.1590/s0036-36342009001000016 20464237

[42] Banks E. Screen-time, obesity, ageing and disability: findings from 91,266 participants in the 45 and up study. Public Health Nutr. 2010; 14(1): 34–43. 10.1017/S1368980010000674 20409356

[43] Durand CP, Gabriel KKP, Hoelscher DM. Transit Use by Children and Adolescents: An Overlooked Source of and Opportunity for Physical Activity?. J Phys Act Health. 2016; 13(8): 861–866. 10.1123/jpah.2015-0444 26999722 PMC5502534

[44] Tetali S, Edwards P, Roberts GMI. How do children travel to school in urban India? A cross-sectional study of 5,842 children in Hyderabad. BMC Public Health. 2016; 16(1): 1099 10.1186/s12889-016-3750-1 27760532 PMC5070088

[45] Merom D, Tudor-Locke C, Bauman A, Rissel C. Active commuting to school among NSW primary schoolchildren: implications for public health. Health & Place. 2006; 12(4): 678–687. 16263323 10.1016/j.healthplace.2005.09.003

[46] Gutiérrez-Zornoza M, Sánchez-López M, García-Hermoso A, González-García A, Chillón P, Martínez-Vizcaíno V. Active Commuting to School, Weight Status, and Cardiometabolic Risk in Children From Rural Areas The Cuenca Study. Health Educ Behav. 2014; 42(2): 231–239. 10.1177/1090198114549373 25228370

[47] Rodríguez-López C, Salas-Fariña ZM, Villa-González E, Borges-Cosic M, Herrador-Colmenero M, Medina-Casaubón J, et al The threshold distance associated with walking from home to school. Health Educ Behav. 2017; 1090198116688429. 10.1177/1090198116688429 28178850

[48] Oliver M, Badland H, Mavoa S, Witten K, Kearns R, Ellaway A, et al Environmental and socio-demographic associates of children’s active transport to school: a cross-sectional investigation from the URBAN study. Int. J. Behav. Nutr. Phys. Act. 2014; 11(1): 70. 24888516 10.1186/1479-5868-11-70 PMC4080694

[49] Oliver M, Parker K, Witten K, Mavoa S, Badland HM, Donovan P, et al Children’s out-of-school independently mobile trips, active travel, and physical activity: a cross-sectional examination from the Kids in the City study. J Phys Act Health. 2016; 13(3): 318–324. 10.1123/jpah.2015-0043 26182349

[50] Stoner L, Matheson A, Hamlin M, Skidmore P. Environmental determinants of childhood obesity: a specific focus on Māori and Pasifika in New Zealand. Perspect Public Health. 2016; 136(1): 18–20. 10.1177/1757913915616734 26702112

[51] CASEN. Informe de estimaciones comunales de pobreza. 2015. Available in: http://observatorio.ministeriodesarrollosocial.gob.cl/documentos/INFORME_estimaciones_pobreza_comunal_2015.pdf

[52] Ross J, Hamlin M. Maori physical activity: a review of an indigenous population's participation. Health promotion journal of Australia. 2007; 18(1), 73–76. 17501715 10.1071/he07073

[53] Lin EY, Witten K, Oliver M, Carroll P, Asiasiga L, Badland H, et al Social and built-environment factors related to children's independent mobility: the importance of neighbourhood cohesion and connectedness. Health & place. 2017; 46, 107–113. 28525801 10.1016/j.healthplace.2017.05.002

[54] Ye X, Wang K, Zou Y, Lord D. A semi-nonparametric Poisson regression model for analyzing motor vehicle crash data. PloS one. 2018; 13(5): e0197338 10.1371/journal.pone.0197338 29791481 PMC5965849

